# Renin-Angiotensin System Genes Polymorphisms and Essential Hypertension in Burkina Faso, West Africa

**DOI:** 10.1155/2015/979631

**Published:** 2015-08-17

**Authors:** Daméhan Tchelougou, Jonas K. Kologo, Simplice D. Karou, Valentin N. Yaméogo, Cyrille Bisseye, Florencia W. Djigma, Djeneba Ouermi, Tegwindé R. Compaoré, Maléki Assih, Virginio Pietra, Patrice Zabsonré, Jacques Simpore

**Affiliations:** ^1^Centre de Recherche Biomoléculaire Pietro Annigoni (CERBA), BP 364, Ouagadougou 01, Burkina Faso; ^2^Laboratoire de Biologie et Génétique Moléculaires (LABIOGENE), Université de Ouagadougou, BP 7021, Ouagadougou 03, Burkina Faso; ^3^Ecole Supérieure des Techniques Biologiques et Alimentaires (ESTBA-UL), Université de Lomé, BP 1515, Togo; ^4^Service de Cardiologie, CHU-Yalgado Ouédraogo, BP 7022, Ouagadougou 03, Burkina Faso; ^5^Centre Médical Saint Camille (CMSC), BP 444, Ouagadougou 09, Burkina Faso; ^6^Laboratoire de Biologie Moléculaire et Cellulaire (LABMC), Université des Sciences et Techniques de Masuku (USTM), BP 943, Franceville, Gabon

## Abstract

*Objective.* This study aimed to investigate the association between three polymorphisms of renin-angiotensin system and the essential hypertension in the population of Burkina Faso. *Methodology.* This was a case-control study including 202 cases and 204 matched controls subjects. The polymorphisms were identified by a classical and a real-time PCR. *Results.* The AGT 235M/T and AT1R 1166A/C polymorphisms were not associated with the hypertension while the genotype frequencies of the ACE I/D polymorphism between patients and controls (DD: 66.83% and 35.78%, ID: 28.22% and 50.98%, II: 4.95% and 13.24%, resp.) were significantly different (*p* < 10^−4^). The genotype DD of ACE gene (OR = 3.40, *p* < 0.0001), the increasing age (OR = 3.83, *p* < 0.0001), obesity (OR = 4.84, *p* < 0.0001), dyslipidemia (OR = 3.43, *p* = 0.021), and alcohol intake (OR = 2.76, *p* < 0.0001) were identified as the independent risk factors for hypertension by multinomial logistic regression. *Conclusion.* The DD genotype of the ACE gene is involved in susceptibility to hypertension. Further investigations are needed to better monitor and provide individualized care for hypertensive patients.

## 1. Introduction

The arterial hypertension is a great public health concern in both industrialized and developing countries. It affects 972 million persons over the world, around one-quarter of the adult population. Among the affected persons, 639 million are living in the developing countries [1]. The arterial hypertension is a multifactorial disease with genetic, environmental, and behavioral determinants [2]. Genetic contribution to blood pressure (BP) variations ranges from 25 to 50% [3].

Among the genetic markers commonly involved, the polymorphisms of several genes, including those encoding vasoactive metabolites such as the components of the renin-angiotensin system (RAS), have been proposed as candidates in association with essential hypertension. The special attention given to the RAS gene polymorphisms is not only due to the fact that its components play an important role in regulation of vascular homeostasis, but also because of the place of angiotensin I converting enzyme (ACE) inhibitors in the therapeutic management of the hypertension.

The polymorphism in exon 2 of the angiotensinogen (AGT) gene, on chromosome 1q42, consists of a substitution of threonine by methionine at position 235 (235M/T). The insertion/deletion polymorphism of the gene encoding the angiotensin I converting enzyme (ACE) consists in the presence (I) or the absence (D) of a 287 bp Alu sequence in intron 16 of this gene on chromosome 17q2. The 1166A/C polymorphism was identified in the 3′ UTR (chromosome 3) of angiotensin II type 1 receptor (AT1R) gene and consists of a substitution of adenosine by cytosine in the messenger RNA. Previous studies focused on the relationship between these polymorphisms and cardiovascular diseases such as hypertension have reported conflicting data [4–7]. In addition, no gene has yet been definitively established to date as responsible for changes in blood pressure, or as a predisposing gene for the hypertension. Thus, the aim of this study was to evaluate the relation between the RAS genes polymorphisms (AGT 235M/T, ACE I/D, and AT1R 1166A/C) and the essential hypertension in the population of Burkina Faso and to determine hypertension main risk factors.

## 2. Materials and Methods

### 2.1. Study Population

The study focused on a population of Burkina Faso (West Africa). This study was approved by the Saint-Camille/CERBA ethical committee. All study participants gave their informed consent and were between 20 and 79 years old. A manual aneroid sphygmomanometer, with armbands adapted to patients and controls morphotypes, was used to measure blood pressure values (systolic and diastolic) by the auscultation method for each subject in a sitting or lying position after at least 10 minutes of rest.

Hypertensive subjects (202) were recruited from the cardiology services in four medical centers of the city of Ouagadougou. The recommendations of scientific societies were used to define and classify the hypertension [8, 9]. A total of 204 matched controls subjects with no cardiovascular disease antecedent were recruited.

Two measurements were made during the same visit, and the retained value was the mean value in the arm with the highest arterial pressure. A standardized questionnaire and clinical examination allowed the collection of data and excluded secondary hypertension and subjects under hormonal treatment to avoid the bias related to the hypertension definition.

Hypertension was defined as BP ≥ 140/90 mmHg. The body mass index (BMI) was determined for each subject by dividing the weight in kg by the square of the height in m^2^. This allowed classifying the subjects into four different groups: subjects of low weight (BMI < 18.5 kg/m^2^), subjects of normal weight (BMI between 18.5 and 24.99 kg/m^2^), overweight subjects (BMI between 25 and 29.99 kg/m^2^), and obese subjects (BMI ≥ 30 kg/m^2^).

The venous peripheral blood was collected from each fasting subject for biochemical and molecular tests. Plasma levels of the following parameters including glucose, total cholesterol, HDL-cholesterol, LDL-cholesterol, and triglycerides were detected using an enzymatic method by the COBAS C311 (Roche-Hitachi, France) automated analyzer.

### 2.2. DNA Extraction and Genotyping

The genomic DNA was extracted from leucocytes using the “DNA Rapid Salting-Out” as described by Miller et al. [10].

### 2.3. Detection of RAS Genes Polymorphisms

The ACE I/D polymorphism (rs4646994) genotyping was performed according to the method described by Rigat et al. [11]. A first PCR was performed with 10 pmol of each primer (sense: 5′-CTG-GAG-ACC-ACT-CCC-ATC-ATT-TCT-3′; antisense: 5′-GTG-GTC-GCC-ATC-ACA-TTG-GTC-AGA-T-3′) in a final volume of 25 *μ*L. Amplification was carried out in 30 cycles with a denaturation phase (94°C for 1 minute), annealing (58°C for 1 minute), and elongation (72°C for 2 minutes) for each cycle and a final extension of 10 minutes at 72°C. Given the fact that the D allele is preferably genotyped with respect to the I allele [12], DD homozygotes were reamplified using the previous protocol [11], with specific primers to the insertion sequence (sense: 5′-TGG-GAC-CAC-AGC-GCC-CGC-CAC-TAC-3′; antisense: 5′-TCG-CCA-GCC-CTC-CCA-TGC-CCA-TAA-3′) [13]. PCR products were electrophoresed on a 2% agarose gel, stained with ethidium bromide, and digitally photographed using a GenFlash instrument.

The AGT 235M/T (rs699) and AT1R 1166A/C (rs5186) polymorphisms were detected by real-time PCR using TaqMan probes on the 7500 Fast Real-Time PCR Systems (Life Technologies, California, USA).

### 2.4. Statistical Analysis

SPSS version 20.0 was used for data analysis. The PowerMarker software Version 3.25 was used for the determination of the Hardy-Weinberg equilibrium and the calculation of allele and genotype frequencies. The Chi-square test (*χ* ^2^) allowed us to compare the differences in the distribution of genotypes and other study variables. Changes were considered statistically significant at *p* < 0.05. Odds ratio (OR) and confidence intervals (CI) at 95% were calculated to estimate the relative risk of hypertension for AGT 235M/T, ACE I/D, and AT1R 1166A/C polymorphisms associated with the continuous categorical variables (age < 50 or ≥50 years, BMI < 25 or ≥25 kg/m^2^, glucose < 6.11 or ≥6.11 mmol/L, total cholesterol < 5.17 or ≥5.17 mmol/L, HDL-cholesterol < 1.68 or ≥1.68 mmol/L, LDL-cholesterol < 2.58 or ≥2.58 mmol/L, and triglycerides < 1.53 or ≥1.53 mmol/L) and discrete variables (sex (M/F), alcohol consumption (yes/no), tobacco consumption (yes/no), lack of physical activity (yes/no), and taking stimulants (yes/no)). The independent predictors of hypertension risk were determined by a multiple logistic regression analysis (forward stepwise method) using HTA status as dependent variable.

## 3. Results

### 3.1. General Characteristics of the Study Population

Table 1 shows the general characteristics of the study population. This population was predominantly female (58.56%). When compared to controls, hypertensive subjects had a similar sex ratio and age but with some significant difference. They were more sedentary, a lot more obese, more hyperglycemic, and more dyslipidemic. Furthermore, they consumed more alcohol, tobacco, and caffeine (coffee, tea, and cola). As it might be expected, their blood pressure values were also high relative to those of controls (*p* < 0.05).

### 3.2. Distribution of Genotypes between Cases and Controls

For each of the three polymorphisms studied (AGT 235M/T, ACE I/D, and AT1R 1166A/C), the genotypes distribution was in Hardy-Weinberg equilibrium for both patients and controls. Allelic and genotypic frequencies of these polymorphisms are presented in Table 2. We had a high frequency for TT genotype (AGT 235M/T), but there were no significant differences in distribution between cases and controls (84.65% versus 86.76%, resp.). The DD genotype and D allele of ACE I/D polymorphism were significantly more frequent in patients than in controls (DD: OR = 3.62, 95% CI = 2.35 to 5.56, *p* < 0.00001; D: OR = 2.68, 95% CI = 1.93 to 3.74, *p* < 0.00001). No homozygous genotype was obtained for the C allele (AT1R 1166A/C), which justifies the null frequencies reported in cases and in controls for this genotype.

We then conducted a stratified analysis to assess the distribution of genotypes of each polymorphism in the different study groups. These results are shown in Table 3. Overall, the same trends as those mentioned in Table 2 emerged. For AGT 235M/T and AT1R 1166A/C polymorphisms, no significant differences were observed between cases and controls. Similarly, difference was observed for the ACE I/D polymorphism between cases and controls.

### 3.3. Risk Factors for Hypertension

Multiple logistic regression analysis (forward stepwise method), using the hypertension status as the dependent variable, including the conventional cardiovascular disease risk factors (sex, smoking, diabetes, obesity, total cholesterol, LDL, HDL, and triglycerides) and new risk factor, such as DD genotype, showed that obesity, increasing age, dyslipidemia, DD genotype, and alcohol intake are independent risk factors for HTA, in decreasing order (Table 4).

## 4. Discussion

The main purpose of this study was to investigate the existence of a possible association between RAS genes polymorphisms and the hypertension and also to identify the main risk factors for this chronic condition in the Burkinabe population. Strong female predominance (58.56%) was found in our study. Yaméogo et al. and Baragou et al. also found similar frequencies in their studies, 56.8% of the hypertensive patients in Burkina Faso [14] and 55.1% of the hypertensive population in urban population in Togo [15], respectively. This dominance could be a simple mass effect or reflect greater susceptibility of the female gender to hypertension among adults. In this study, the median age for a hypertensive was 51 ± 10.01 years. This age is close to the 48.96 ± 12.99 years reported by Yayehd et al. in Togo [16] but was higher than the 33.1 ± 13.3 years reported by Niakara et al. in Burkina Faso [17].

The age of study population varied from 20 to 79 years. This is because there was an important proportion of young subjects (under 50 years) among hypertensives, and so we needed to have young subjects in controls group to match cases. However, the young age of some controls may constitute a limitation of this study. Indeed, among the young controls, some may be hypertensive in the future skewing the selection of controls subjects.

The different approaches used in studies focused on genetic susceptibility to disease in African populations have been previously described by Sirugo et al. [18]. Among the approaches used for searching for hypertension loci, few are linkage studies and most often are association studies [19]. The use of candidate gene approach includes the renin-angiotensin system genes but given the ethnic diversity and the selection bias, the results from these studies are often faced with interpretation difficulties [20–23]. The uniqueness of this study lies in the fact that it is the first of its kind in the Burkinabe population that combines the RAS genes polymorphisms and the hypertension. Furthermore, selection bias was minimized by overlapping controls to cases for sex and age variable, which are nonmodifiable risk factors.

Jeunemaitre et al. reported an association between the TT genotype of the AGT gene and the risk of hypertension in Utah and France [24]. Subsequently, cases of associations between the same genotype TT and the risk of hypertension in other populations have been reported [20, 21, 25, 26]. Nevertheless, we did not find any evidence of association between AGT 235M/T polymorphism and the incidence of hypertension in our study population. Similarly, Rotimi et al. [6] and Tiago et al. [23] found no association between AGT 235M/T polymorphism and the hypertension in their study. But compared to the previous work, we obtained a higher frequency of the TT genotype in this study.

The DD genotype of the ACE gene was present in 35.78% of controls, compared to 66.83% of hypertensive patients. This result showed a strong association between the ACE I/D polymorphism and the risk of hypertension in the Burkinabe population (*p* < 0.0001). Mehri et al. [26] and Jiménez et al. [27] also reported strong associations between the DD genotype and the risk of hypertension in their study populations. However, Castellano et al. [28] did not find any evidence of association between the D allele and the risk of hypertension in the Italian population. Thus, the DD homozygous persons have an increased risk, almost four times, of developing hypertension in the Burkinabe population compared with homozygous I allele carriers. In other studies, a positive association between the ACE I/D polymorphism and obesity is also reported [26]. However, in this work, we had found no association between the ACE I/D polymorphism and obesity (data not shown), suggesting that this polymorphism is independent of the body mass levels as it is related to the risk of hypertension. The logistic regression analysis confirmed that the DD genotype of the ACE gene is a risk factor that would increase by nearly 4 times the incidence of hypertension among patients, regardless of other environmental risk factors. Moreover, Kato et al. [29] had found that the DD genotype of the ACE I/D polymorphism is a major risk factor for cerebral and cardiovascular events like stroke in Japanese hypertensive patients.

The potential effects of AT1R 1166 A/C polymorphism in the predisposition to hypertension are not well understood. Bonnardeaux et al. had initially found high prevalence of the allele 1166C in hypertensive subjects compared to what they found in normotensive subjects [5]. Recently, Mehri et al. reported an association between this polymorphism and hypertension in Tunisian patients with type 2 diabetes [26]. In our study, not only was there no association observed between the AT1R 1166C allele and hypertension, but we did not find homozygous genotype for this allele, both in cases and controls. The lack of association has also been reported in other populations [30]. These observations should be therefore interpreted with some caution.

Our results showed a strong association between the ACE I/D polymorphism and the development of hypertension. Thus, DD genotype is a predictor of hypertension risk, independent of other environmental factors. This work has also shown that obesity prevalence was 11% among controls and was the major risk factor of hypertension among the Burkinabe population (OR = 4.84, 95% CI = 2.96 to 7.92; *p* < 0.0001). This obesity frequency was twice as high in women as in men (*p* < 0.001; data not shown). The high prevalence of obesity in developing countries is related to the socioeconomic level of the population [31], along with the adoption of harmful eating behaviors, including snacking, a diet low in fiber and high in fat. In addition, the high prevalence of overweight in women is due to their physical inactivity and cultural conceptions [32].

For the three polymorphisms studied, we find evidence of the association with hypertension risk only for the ACE I/D polymorphism. However, Niu et al. [20] and Mehri et al. [26] had reported, respectively, in Chinese and Tunisian populations, a positive association between all the three polymorphisms and hypertension.

The reported discrepancies reflect genetic, cultural, and ethnic diversity within populations of study, people who mostly live in different environmental contexts, and are therefore exposed to various problems. Thus, the negative associations reported may be due to these differences and possible sampling bias [33]. In addition, the markers studied could be linked to other genes with which they have an impact on hypertension incidence in the population or on other disease events. This reinforces the idea of studying several polymorphisms in different ethnic groups to have good appreciation [34].

## 5. Conclusion

AGT 235M/T and AT1R 1166A/C polymorphisms showed no association with the risk of developing hypertension. However, the ACE gene DD genotype predisposes to the occurrence of hypertension in the Burkinabe population. Other major risk factors identified are obesity, advancing age, dyslipidemia, and alcohol consumption. ACE I/D polymorphism can serve as a marker for early diagnosis of hypertension if we have similar findings elsewhere. More studies involving ACE I/D polymorphism with hypertension therapeutic responses are still needed to better monitor and provide individualized care for hypertensive patients.

## Figures and Tables

**Table 1:** General characteristics of study population (cases versus controls).

Characteristics	Controls	Cases	*p* value
Total	**204**	**202**	
Sex ratio (M/F)	85/119	84/118	0.99
Age, years^#^	49.50 ± 13.54 (20–78)	51 ± 10.01 (21–76)	0.205
BMI, Kg/m²^#^	23 ± 4.90	27 ± 6.48	<0.00001
SBP, mmHg^#^	120 ± 11.47	160 ± 20.66	<0.00001
DBP, mmHg^#^	70 ± 8.24	95 ± 11.87	<0.00001
PP, mmHg^#^	40 ± 10.29	70 ± 16.75	<0.00001
Glycemia, mmol/L^#^	3 ± 1.70	5 ± 2.34	<0.00001
Total-C, mmol/L^#^	4 ± 1.54	5 ± 1.29	<0.00001
HDL-C, mmol/L^#^	1 ± 0.43	1 ± 0.59	NA
LDL-C, mmol/L^#^	2 ± 1.17	3 ± 1.13	<0.00001
Triglycerides, mmol/L^#^	1 ± 0.74	1 ± 0.61	NA
Hyperglycemia, %	8.51	21.05	0.000236
Dyslipidemia, %	51.06	77.27	<0.00001
Obesity, %	11.76	30.69	0.000003
Alcohol intake, %	22.55	46.53	<0.00001
Smoking, %	6.37	11.39	0.07559
Excitant intake, %	42.16	46.53	0.3746
Sedentary, %	14.22	17.82	0.3218

^#^Median ± SD for continuous variables; NA: not applicable.

BMI: body mass index; SBP: systolic blood pressure; DBP: diastolic blood pressure; PP: pulse pressure; Total-C: total cholesterol; HDL-C: high density lipoprotein cholesterol; LDL-C: low density lipoprotein cholesterol; Trigly: triglycerides.

**Table 2:** Frequencies of renin-angiotensin system genes polymorphisms (cases versus controls).

Polymorphisms	Controls	Cases	OR	95% CI	*p* value
Total	**204**	**202**			
*AGT* 235M/T					
TT versus MT + MM, *n* (%)	177 (86.76)	171 (84.65)	0.84	0.46–1.53	0.57
MT versus TT + MM, *n* (%)	24 (11.77)	29 (14.36)	1.26	0.68–2.35	0.46
MM versus TT + MT, *n* (%)	3 (1.47)	2 (0.99)	0.67	0.06–5.92	1
T versus M, *n* (%)	378 (0.93)	371 (0.92)	0.89	0.51–1.54	0.70
HWE *p* value	**0.15**	**0.83**			
*ACE* I/D					
DD versus ID + II, *n* (%)	73 (35.78)	135 (66.83)	3.62	2.35–5.56	**<0.00001**
ID versus II + DD, *n* (%)	104 (50.98)	57 (28.22)	0.38	0.25–0.58	<**0.00001**
II versus DD + ID, *n* (%)	27 (13.24)	10 (4.95)	0.34	0.14–0.76	**0.005**
D versus I, *n* (%)	250 (0.61)	327 (0.81)	2.68	1.93–3.74	**<0.00001**
HWE *p* value	**0.57**	**0.48**			
*AT1R* 1166 A/C					
CC versus AC + AA, *n* (%)	0 (0.00)	0 (0.00)	0	0–4.21	0.19
AC versus AA + CC, *n* (%)	7 (3.43)	7 (3.47)	1.01	0.30–3.44	1.00
AA versus CC + AC, *n* (%)	197 (96.57)	195 (96.53)	0.99	0.29–3.38	1.00
C versus A, *n* (%)	7 (0.02)	7 (0.02)	1.01	0.30–3.41	1.00
HWE *p* value	**0.97**	**0.97**			

OR: odds ratio; CI: confidence intervals; HWE: Hardy-Weinberg equilibrium; *AGT*: angiotensinogen; *ACE*: angiotensin converting enzyme; *AT1R*: angiotensin II type 1 receptor.

**Table 3:** Genotypes distribution among study population groups.

Groups	Genotypes									Total
	*AGT* 235M/T			*ACE* I/D			*AT1R* 1166A/C			
	TT	MT	MM	DD	ID	II	CC	AC	AA	
Males										
Controls	91.8%	7.1%	1.2%	36.5%	51.8%^*∗∗*^	11.8%	0.0%	2.4%	97.6%	**85**
Cases	82.1%	16.7%	1.2%	69.0%^*∗∗*^	25.0%	6.0%	0.0%	4.8%	95.2%	**84**
Females										
Controls	83.2%	15.1%	1.7%	35.3%	50.4%^*∗*^	14.3%^*∗*^	0.0%	4.2%	95.8%	**119**
Cases	86.4%	12.7%	0.9%	65.3%^*∗∗*^	30.5%	4.2%	0.0%	2.5%	97.5%	**118**
<50 years										
Controls	87.1%	10.9%	2.0%	34.0%	53.1%^*∗∗*^	12.9%	0.0%	3.4%	96.6%	**147**
Cases	83.5%	15.2%	1.3%	67.1%^*∗∗*^	26.6%	6.3%	0.0%	3.8%	96.2%	**79**
≥50 years										
Controls	86.0%	14.0%	0.0%	40.4%	45.6%^*∗*^	14.0%^*∗*^	0.0%	3.5%	96.5%	**57**
Cases	85.4%	13.8%	0.8%	66.6%^*∗∗*^	29.3%	4.1%	0.0%	3.3%	96.7%	**123**
<25 kg/m²										
Controls	87.1%	11.6%	1.3%	36.7%	49.0%^*∗*^	14.3%^*∗*^	0.0%	4.1%	95.9%	**147**
Cases	86.7%	12.0%	1.3%	66.7%^*∗∗*^	29.3%	4.0%	0.0%	2.7%	97.3%	**75**
≥25 kg/m²										
Controls	86.0%	12.3%	1.7%	33.3%	56.2%^*∗∗*^	10.5%	0.0%	1.8%	98.2%	**57**
Cases	83.5%	15.7%	0.8%	66.9%^*∗∗*^	27.6%	5.5%	0.0%	3.9%	96.1%	**127**

^*∗*^ *p* < 0.05; ^*∗∗*^ *p* < 0.001.

**Table 4:** Multinomial logistic regression analysis for hypertension risk factors.

	OR	95% CI	*p* value
Age	**3.83**	**2.32–6.32**	**<0.0001**
Sex	1.19	0.55–2.59	0.664
Obesity	**4.84**	**2.96–7.92**	**<0.0001**
DD	**3.40**	**2.1–5.5**	**<0.0001**
Smoking	0.52	0.108–2.48	0.410
Alcohol intake	**2.76**	**1.65–4.61**	**<0.0001**
Sedentary	0.15	0.017–1.23	0.077
Excitant intake	0.48	0.27–0.85	0.012
Hyperglycemia	1.52	0.34–6.87	0.586
Dyslipidemia	**3.43**	**1.21–9.79**	**0.021**

OR: odds ratio; CI: confidence intervals; DD: DD genotype of ACE I/D polymorphism.
